# Musical Experience, Auditory Perception and Reading-Related Skills in Children

**DOI:** 10.1371/journal.pone.0075876

**Published:** 2013-09-24

**Authors:** Karen Banai, Merav Ahissar

**Affiliations:** 1 Department of Communication Sciences and Disorders, University of Haifa, Haifa, Israel; 2 Departments of Psychology and Cognitive Sciences, Hebrew University, Jerusalem, Israel; UNLV, United States of America

## Abstract

**Background:**

The relationships between auditory processing and reading-related skills remain poorly understood despite intensive research. Here we focus on the potential role of musical experience as a confounding factor. Specifically we ask whether the pattern of correlations between auditory and reading related skills differ between children with different amounts of musical experience.

**Methodology/Principal Findings:**

Third grade children with various degrees of musical experience were tested on a battery of auditory processing and reading related tasks. Very poor auditory thresholds and poor memory skills were abundant only among children with no musical education. In this population, indices of auditory processing (frequency and interval discrimination thresholds) were significantly correlated with and accounted for up to 13% of the variance in reading related skills. Among children with more than one year of musical training, auditory processing indices were better, yet reading related skills were not correlated with them. A potential interpretation for the reduction in the correlations might be that auditory and reading-related skills improve at different rates as a function of musical training.

**Conclusions/Significance:**

Participants’ previous musical training, which is typically ignored in studies assessing the relations between auditory and reading related skills, should be considered. Very poor auditory and memory skills are rare among children with even a short period of musical training, suggesting musical training could have an impact on both. The lack of correlation in the musically trained population suggests that a short period of musical training does not enhance reading related skills of individuals with within-normal auditory processing skills. Further studies are required to determine whether the associations between musical training, auditory processing and memory are indeed causal or whether children with poor auditory and memory skills are less likely to study music and if so, why this is the case.

## Introduction

Auditory processing has been proposed to play a role in the development of reading (e.g., [1,2]), because learning to read requires linking the sounds of spoken language with their written forms (see 3). Nevertheless, the role of auditory processing in reading and reading-related language skills remains debated despite the many studies attempting to clarify it. One source of difficulty is the inconsistency of findings across different studies. Although many studies reported significant correlations between reading-related skills and auditory skills (e.g., [1,2,4-17]), others failed to find such correlations [18-25]. More specifically, whereas in several studies, pitch processing was found to account for significant variance in reading skills (e.g., [1,9,17]), this was not the case in other studies (e.g., [25,26]). This discrepancy was attributed to differences in the types of perceptual tasks used [27-29] or to differences in the characteristics of the sampled populations [12,30]. Here, we consider an additional factor - the potential contribution of musical experience to the relationships between auditory processing and reading-related skills. Specifically, we ask whether musical training affects both auditory processing and reading related skills in a similar manner, and with similar time constants.

It is well documented that individuals with long-term musical experience have better auditory skills than non-musicians (reviewed in 31). Musicians’ performance is better than that of individuals with no musical experience in analyzing both trained and untrained acoustic features. They are better in discriminating trained melodic relationships, and in fine-grained discrimination of basic acoustic features such as pitch [32,33] and duration [34,35]. Those perceptual advantages are accompanied by group differences in cortical (e.g., [36] and see 37 for review) and subcortical (e.g., [38-40]) processing of sound. A longitudinal study in which children were pseudo-randomly assigned to music vs. non-music groups suggests that even relatively short-term musical training (~one year) results in enhanced behavioral sensitivity to small pitch variations in speech [41]. It seems that such training also induces modifications in the neural encoding of sound because greater changes in electrophysiological indices of auditory function were found in children taking music lessons than in age matched children not taking such lessons [42,43]. Studies in which individuals with different amounts of musical training were compared suggest that even individuals with relatively short-term musical experience have enhanced auditory processing in a musical context when compared with non-musicians [44-47], though the extent of generalization to pure tone discriminations was not directly assessed.

In contrast to the relatively clear effects of musical experience on auditory processing, the effects of musical training on other cognitive and language skills are still debated [44,48,49]. For example, longitudinal studies suggest that a few years of musical training can positively contribute to verbal (but not visual) memory [50,51], and to reading of inconsistent (but not regular) words [41]. More refined neural processing of linguistic pitch patterns in tonal languages is found in musicians, even when they are not speakers of a tonal language [52,53]. On the other hand, a meta-analysis of 6 studies, in which children were randomly assigned to groups that either received or did not receive music lessons, failed to find a consistent effect of music lessons on indices of reading performance [48].

More consistent broader benefits putatively associated with musical experience were shown when individuals with intensive long-term musical training (i.e. musicians) were assessed. In addition to enhanced auditory perception [33,34,54], studies report enhanced sensory-motor [55-57], linguistic [58] and cognitive [44,50,59-61] skills. These are accompanied by changes in brain function (see 31,62,63 for reviews). Though causality is typically not directly shown, the common finding that the magnitude of these benefits is correlated with the number of years of musical training and is larger with an earlier onset of practice supports this interpretation. Nevertheless, the alternative, that more motivated or more talented individuals start playing at an earlier age and continue to play longer than less motivated/talented ones, cannot be ruled out because randomly assigned groups were not (yet) followed long-term.

Thus, while the literature suggests that the impact of prolonged musical practice is partially generalized to other cognitive skills, the dynamics of this generalization is far from understood. Perhaps auditory perception, which is tightly linked with musical training, is immediately improved by musical practice whereas linguistic skills, which are related only indirectly, improve only subsequently, in a cascade manner. If musical training has a larger and/or faster influence on auditory perception than on reading-related cognitive skills, it will at least initially, reduce the observed correlations between these two domains. Yet, to the best of our knowledge, musical experience was not considered in any of the above mentioned studies. In fact, we are aware of only one study that assessed the possible influence of musical training on the pattern of correlations between auditory and reading skills [64]. In this study, pitch, rhythm and timbre discrimination were assessed in addition to phonological awareness and word identification skills. It was found that pitch discrimination was correlated with phonological awareness and with accuracy of single word reading in children with no musical experience, but not among children with an average of 2 years of musical experience. This study suggests that musical experience may indeed initially reduce the observed pattern of correlations between auditory perception and reading related skills. However, given the relatively small group of musically trained children (n=26), their broad range of musical education (0.5-5 years) and their broad age range (5-9 years old), its findings are mainly suggestive. To study these relationships systematically, we recruited a community sample of 184 children from a restricted age range (third grade). We characterized auditory discriminations (frequency and temporal-interval), memory and reading skills as a function of the amount of formal musical education (none to 3 years). We then separately calculated the correlations between these skills in groups with different amounts in musical training.

## Materials and Methods

### 1: Participants

184 third-grade children (mean age 8; 8±0; 4) from three mainstream public schools participated in this study. Based on parent and teacher reports children were typically developing with no history of hearing or neurological problems. Fifteen children failed to provide information about their musical background and were therefore excluded from the study. Additional 13 children failed to complete parts of the testing protocol due to time constraints or technical problems, and their data were excluded from data analysis. Of the remaining 156 children, 108 were taking music lessons at the time of the study and are thus referred to as ‘musically experienced’. The remaining children never participated in any form of formal music lessons and are thus referred to as ‘musically naïve’. ‘Musically experienced’ children were taking formal music lessons for 1-36 months (mean 13±9 months). Musically experienced children were taking weekly music lessons, learning to play an instrument -- most typically piano or recorder

The study was approved by the ethics committee of the Department of Psychology of the Hebrew University. Data collection in schools was approved by the chief scientist in the Ministry of Education and was conducted according to their guidelines. Written consent was obtained from the parents of all children prior to data collection.

### 2: Stimuli and Tasks

#### Auditory psychophysical tasks

Frequency and temporal-interval discrimination were assessed using adaptive (3-down/1-up) auditory discrimination tasks designed to track the 79% correct point on the psychometric function. Each procedure began with an oral explanation, followed by 10 easy (using only the largest inter-tone difference) practice trials. If the participant performed less than 8/10 correct, he/she was given 10 additional training trials. The subsequently administered assessment blocks, from which thresholds were computed, were 80 trials long.

*Frequency Discrimination.* Two 200 msec pure tones were presented in each trial and children had to determine which one was higher – the first or the second. The first tone (the reference) was always 1000 Hz, whereas the frequency of the second tone (the test tone) was adapted based on the responses of the listener. The initial frequency difference between the reference and the test tones was 50%. Subsequently the frequency difference decreased/increased in a 3-down/1-up staircase. During the staircase procedure, step size was decreased every 4 reversals, as follows: For the first 4 reversals the frequency differences were halved/doubled; subsequently the frequency difference was divided/multiplied by 1.4, 1.1 and 1.05. This task was completed by a total of 156 children, of whom 108 were taking music lessons at the time of the study.

*Temporal-Interval Discrimination.* Two pairs of 50-msec 1-kHz tones were played on each trial and children had to select the pair defining the longer interval- the first or the second. The first interval (the reference) was always 375 msec (offset to onset), whereas the second (the test) interval was adapted based on the responses of the listener, beginning with a 50% difference. On subsequent trials, the duration of the test interval was adapted in a procedure identical to that described for the frequency discrimination task. This task was completed by 108 of the children who completed the frequency discrimination task, 68 of whom were taking music lessons at the time of the study.

The tasks were administered through the built-in soundcard of a laptop and circumaural headphones (JTS HP-535). A graphical interface created in Flash was used to present the sounds, run the adaptive procedure and collect listeners’ responses. To experience the interface, visit: http://papi.huji.ac.il/Default.aspx. In both tasks inter-stimulus interval was 1000 msec. Listeners could make their response as soon as the second tone ended and there was no time limit on the response interval. Inter-trial-interval was 500 msec from the response made by the subject.

*Calculation of discrimination thresholds (Just Noticeable Difference, JND*)*.* Participants in this study had 9-19 reversals in each block of 80 trials. These numbers are consistent with our previous studies in both children and adults [12,28,65,66]. Therefore, raw JNDs were calculated as the mean frequency or duration differences between the reference and non-reference intervals in the last 7 reversals of each block. JNDs were log transformed so that a discrimination threshold of 1% (i.e. a fraction of 0.01) translates to a log value of -2, whereas a discrimination threshold of 10% translates to a log value of -1. Log transforms were used to normalize the distribution of JNDs and allow for the use of parametric statistics.

#### Reading

Decoding pointed Hebrew words and pseudo-words was tested using the lists developed by Deutsch and Bentin [67]. Reading accuracy was defined as the mean percent correct across the two lists; reading duration was defined as the average reading duration (in seconds) across the two lists.

#### Reading-related skills

*Verbal Memory* was assessed using the standard Digit span task (Wechsler, 1998; Israeli edition [68]). In this task, participants listen to increasingly longer lists of digits read by the experimenter and repeat them in order of presentation (Digit forward) or in reversed order (Digit backwards). The number of correctly recalled lists in the forward part was used as an index of memory span. The total number of correctly recalled lists in the backward subtest was used as an index of working memory.

*Phonological Awareness.* A (CV or CVC) syllable deletion task containing 12 items was used. Participants were read a word and were asked to produce the word without a specific syllable, the first (4 items), the middle (5 items), or the last one (3 items) (e.g. "/Ra-ashan/ without /Ra/" -> /Ashan/).

#### General reasoning abilities

General reasoning abilities were assessed using Raven’s Standard Progressive Matrices [69]. In this test, arrays of visual objects, each with a missing one, are presented. Participants are asked to complete each array by selecting from six alternatives.

### 3: Testing procedure

Children were tested individually, in a quiet room in each school. Two sessions, each lasting 45 minutes (one school period) were administered to each participant few days apart. Children were tested with psychophysical and non-psychophysical tasks in an interleaved manner. On the first session children were tested on frequency discrimination, Raven’s matrices and word reading. Temporal-interval discrimination, non-word reading, verbal memory and phonological awareness were tested on the second session.

### 4: Data analysis

An inspection of the skewness and kurtosis of all the variables within our data set suggested that the distributions were approximately normal. Therefore, parametric statistics were used in all subsequent analyses. Pearson and partial correlations were used to evaluate the relationships between indices of auditory processing on the one hand and reading-related skills on the other. To determine whether children with different amounts of musical experience differed on any of the auditory processing or reading-related measures, children were divided to groups with different amounts of musical experience (see Sections 3.2 and 3.3 below), and planned comparisons (contrasts) were use to compare each of the groups with musical experience to the ‘no experience’ group. With the exception of Raven’s matrices, variance in all other variables was homogeneous across groups (Levene’s statistic < 2.2, p > 0.09).

## Results

### 1: Frequency discrimination, verbal memory and reading-related skills

Modest but significant correlations were observed between frequency discrimination on the one hand and reading related measures on the other hand (n=156). These include word reading, verbal memory and phonological awareness (Table 1, left columns). A similar pattern of correlations was found between temporal-interval discrimination and reading related measures (a subgroup of 108 children; Table 1, right columns). Both types of auditory thresholds were more highly correlated with measures of verbal memory, which, unlike reading and phonological awareness, is not directly taught in schools. Auditory thresholds, as well as verbal memory and phonological awareness scores were also correlated with non-verbal visual reasoning abilities (as measured by the Raven matrices). Still, as shown in Table 1, the use of partial correlations to account for the statistical contribution of Raven matrices to the observed correlations did not substantially alter the pattern of correlations between auditory and reading-related measures. This indicates that the associations between perceptual and language skills are not a mere outcome of the contribution of general abilities to performance in both domains.

**Table 1:** Correlations between auditory discrimination, cognitive and literacy related skills.

	Frequency discrimination (log JND) N=156		Temporal-interval discrimination (log JND) N=108		
	Pearson	Partial (controlling for Raven)		Pearson	Partial (controlling for Raven)
Reading accuracy(% correct)	-0.21**	-0.16*		-0.17	-0.14
Reading rate (duration of reading the list)	0.18*	0.18*		-0.03	-0.05
Memory span (digit forward)	-0.33***	-0.22**		-0.34***	-0.24*
Working memory (digit backward)	-0.20*	-0.08		-0.39***	-0.29**
Phonological awareness (accuracy)	-0.26**	-0.17*		-0.29**	-0.24*
Cognitive skill (Raven)	-0.36***	−		-0.33**	−

*p < 0.05; **p < 0.01; ***p < 0.001

### 2: The relationships between musical experience and task performance

General cognitive ability, verbal memory span, frequency and temporal-interval discrimination thresholds were also significantly correlated with musical experience (quantified as time since the beginning of formal music lessons) of the children in our sample (Pearson correlations: Raven: r = 0.24, p = 0.003; Digit forward: r = 0.23, p = 0.004; Frequency discrimination: r = -0.34, p < 0.001; Temporal-interval discrimination: r = -0.22, p = 0.022).

**Figure 1:**
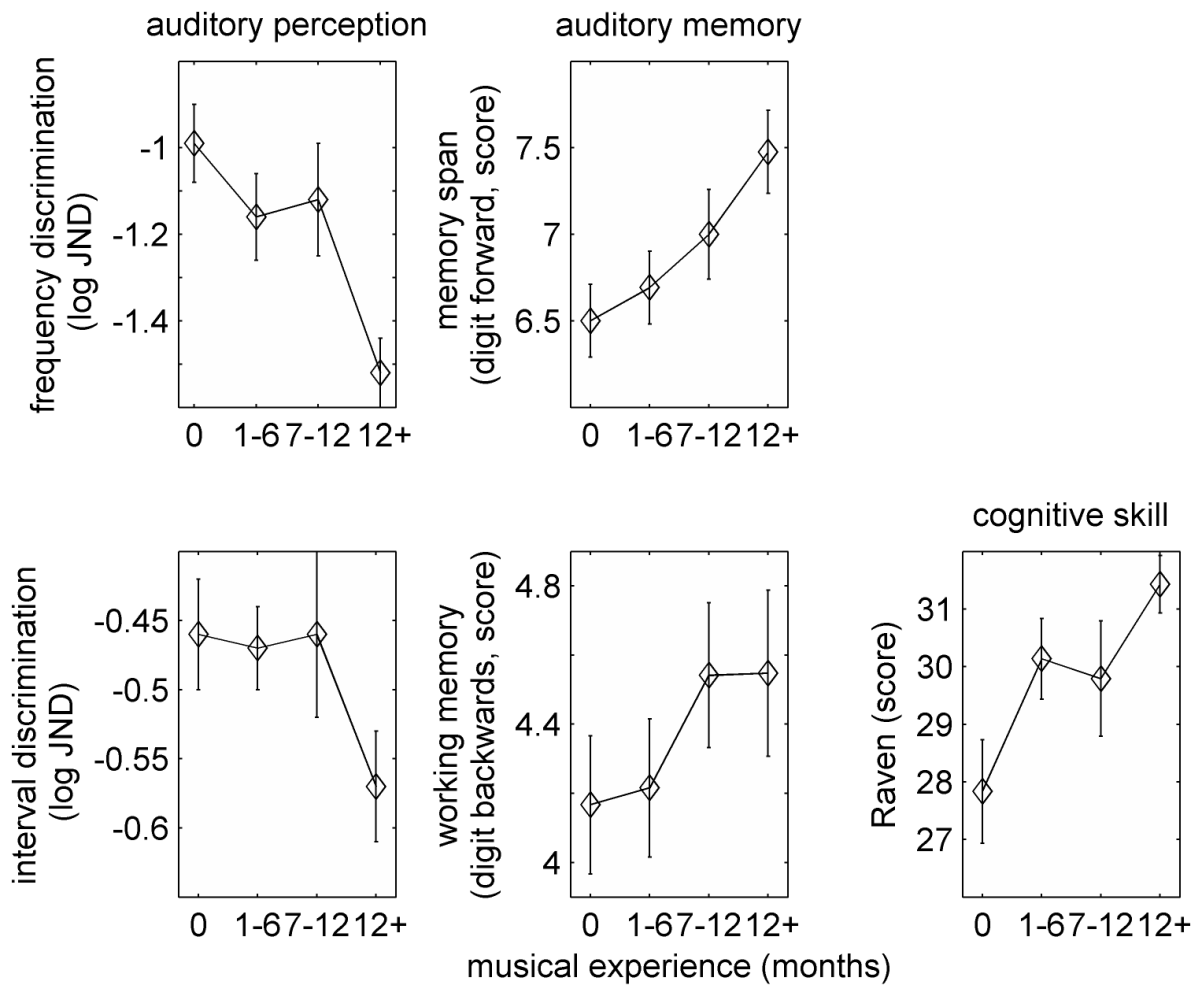
Auditory perception (frequency and interval discrimination), verbal memory (Digit forward and Digit Backward) and reasoning ability (Raven’s matrices) as a function of musical experience, in months of formal training. Error bars are ± 1 standard error of the mean.

To take a closer look at the differences between children with different amounts of musical experience, the 156 children who completed both frequency discrimination and the reading and reading-related tasks were divided into 4 groups based on the time they were taking music lessons: one group (n = 48) never engaged in any formal music training. The remaining groups had 1-6, 7-12 or more than 12 months of music lessons (n = 45, 22 and 41, respectively). Analyses of variance with musical experience as a between subject factor revealed significant group differences in frequency discrimination (F(3,152) = 5.64, p = 0.001), memory span (F(3,152) = 3.16, p = 0.026) and Raven scores (F(3,152) = 4.16, p = 0.007), as illustrated in Figure 1. Other measures, like working memory, phonological awareness and reading rate and accuracy, were not significantly different between the groups. Contrast analyses comparing each of the groups with musical experience to the ‘no experience’ group show that the group with more than 1-year of musical experience had significantly lower (better) frequency discrimination thresholds (t(152) = 4.04, p < 0.001, Cohen’s d = 0.86, Figure 1, top left), longer memory spans (t(152) = -3.03, p = 0.003, Cohen’s d = 0.65, Figure 1, top, second panel from left) and higher Raven scores (t(73.24) = -3.64, p < 0.001, Cohen’s d = 0.61). In fact, even the group with only 1-6 months of musical training had higher Raven scores than the ‘no experience’ group (t(88.51) = -1.91, p = 0.03; see Figure 1, bottom row, rightmost panel).

A similar analysis was conducted on the temporal interval discrimination thresholds of the 108 children who completed this task (Figure 1, bottom, left). Although the omnibus ANOVA was nonsignificant, the planned contrast analysis suggested that the group with more than 1 year of musical experience tended to have lower thresholds than the ‘no experience’ group (t(108) = 1.95, p = 0.054), albeit with a small effect size (Cohen’s d = 0.36).

The current data are consistent with previous studies that reported differences in IQ as a function of musical experience. Generally, IQ differences in relation to musical experience are attributed to the fact that children with higher IQs are more likely to study music rather than to causal effects of training [70]. Indeed, the difference in Raven scores between children with and without musical experience makes it hard to determine whether other differences are simply attributable to pre-existing IQ differences that make some children more likely than others to study music and to have better perceptual and memory skills. To account for these effects, the group analysis was repeated while statistically accounting for IQ differences by using Raven scores as covariates. This analysis left the group differences in frequency discrimination significant (F(3,152) = 3.46, p = 0.018), but obliterated the effects on memory spans (F(3,152) = 1.91, p = 0.131). Therefore, it appears that whereas the effect of short-term musical experience on auditory frequency discrimination cannot be fully attributed to differences in general reasoning skills, the longer memory spans in musically trained children (at least with the limited experience of the children in the current study) may reflect broader, pre-existing IQ differences.

### 3: The pattern of correlations between auditory discrimination and reading-related skills among children with different amounts of musical experience

The correlations between frequency and temporal-interval discrimination on the one hand and reading related skills on the other hand, were calculated separately for children with no musical experience (n=40), and for children with more than one year of musical experience (n=26).

**Figure 2:**
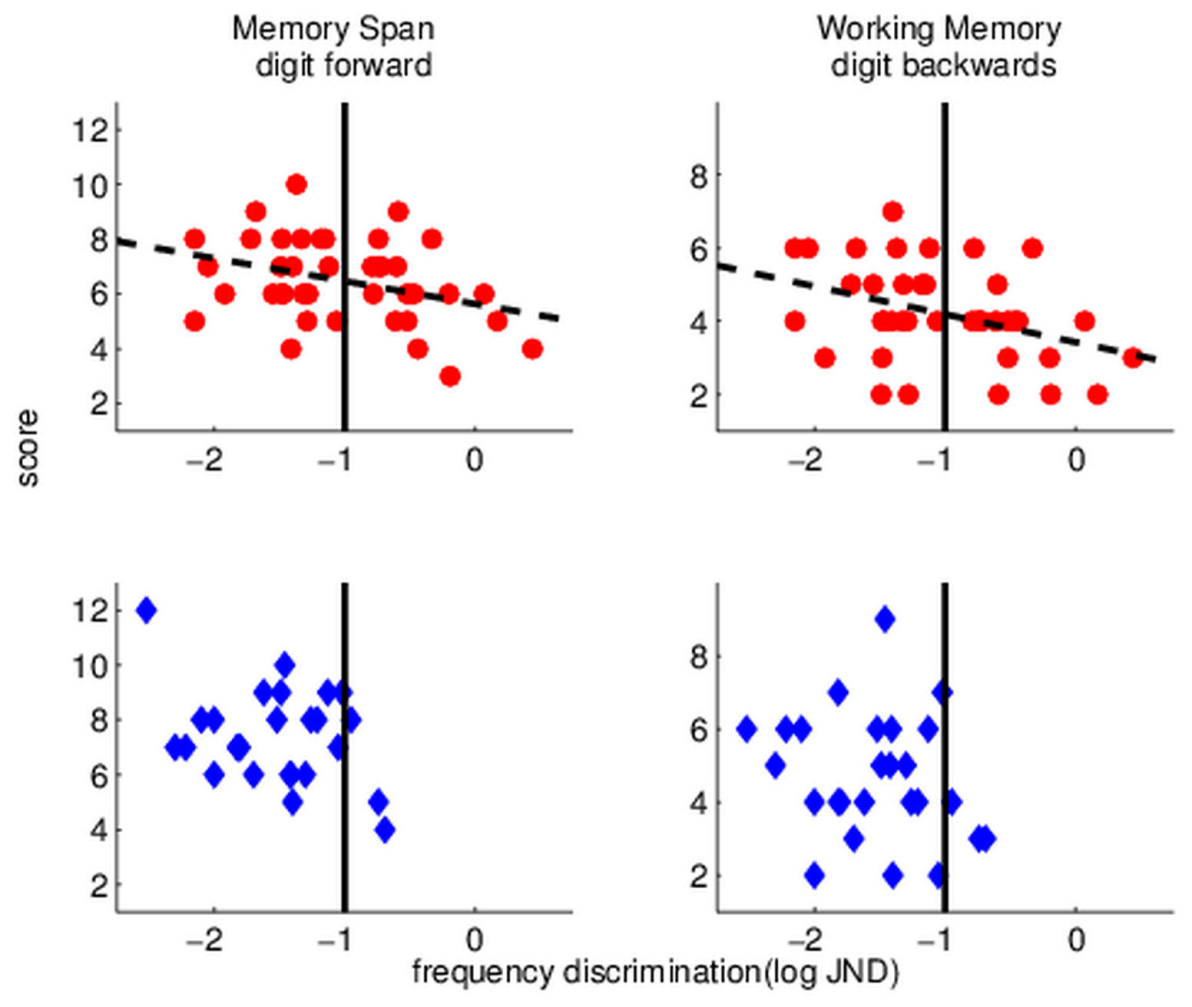
Scatter plots illustrating the relationships between frequency discrimination and two aspects of verbal memory, simple span (Digit forward; left column) and working memory span (Digit backward; right column) in musically naïve (top panels) and in musically experienced (more than a year; bottom panels) children. Dashed lines show the linear relationships between memory and frequency discrimination. Note that among musically trained children the JNDs of only 3/26 children were poorer than 10% (corresponding to a value of -1 in log units) whereas among musically naïve individuals 18/40 had these poor thresholds. In both groups these individuals tend to have poor memory scores.

**Figure 3:**
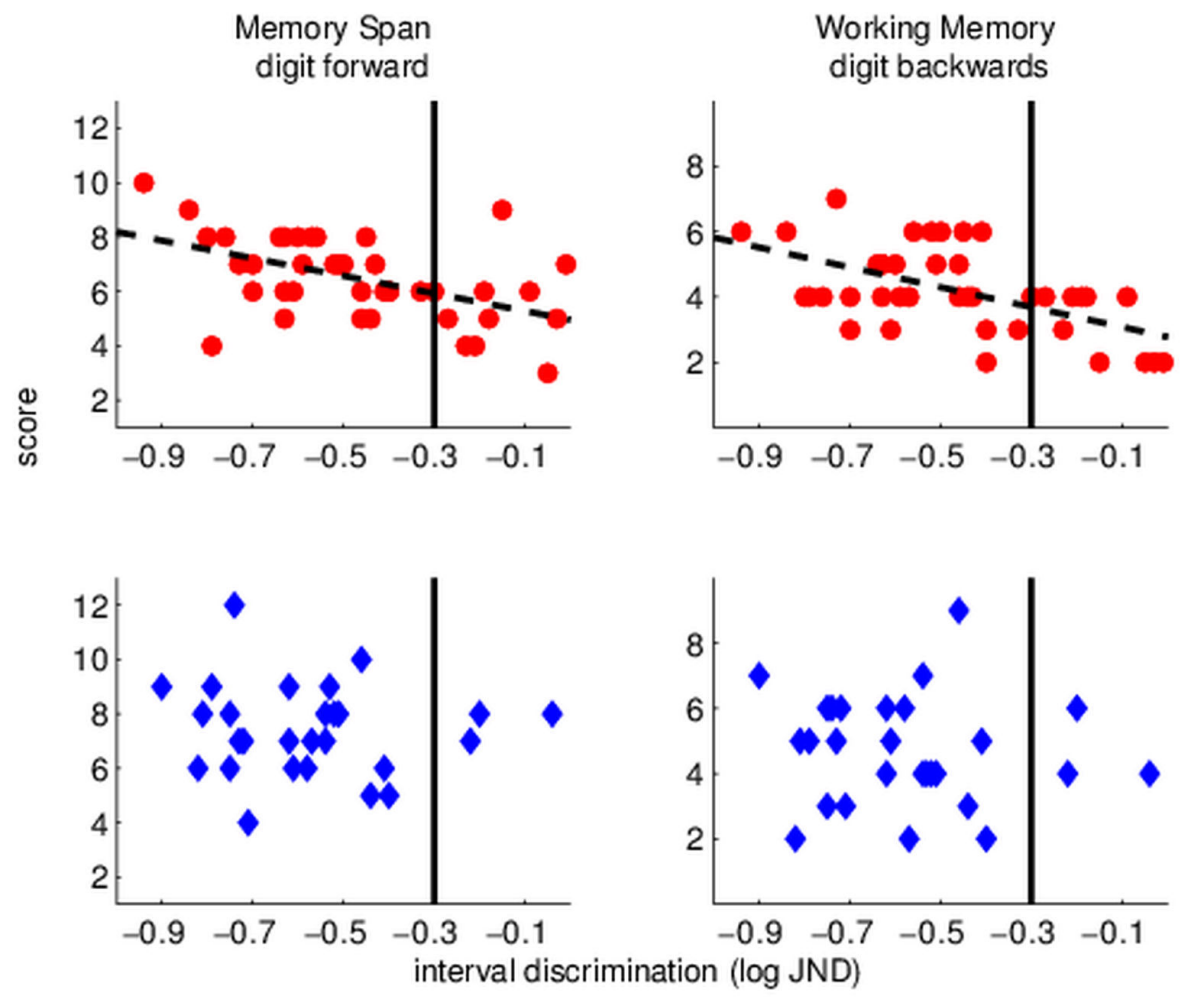
Scatter plots illustrating the relationships between temporal-interval discrimination and two aspects of verbal memory, simple span (Digit forward; left) and working memory (Digit backward; right) in musically naïve (top panels) and musically experienced (more than a year of training; bottom panels) children. Among musically naïve children 10/40 have poor (> 50%) thresholds (right of the vertical lines denoting JND = -0.3). These children tend to have poorer memory spans, particularly poor working memory span (top right panel).

Figure 2 shows memory spans (left) and working memory (right) as a function of frequency discrimination in the groups with (bottom) and without (top) musical experience. The scatter plots suggest a different distribution of performance between the groups. Most salient is the difference in frequency discrimination thresholds. Among individuals with no musical background the proportion of very poor performers (denoted by the vertical line; thresholds > -1, i.e. larger than 10%) is much higher than that among individuals with more than one year of training (Fisher’s exact test: p = 0.001). Importantly, these participants, with no musical experience and poor frequency discrimination tend to have lower memory spans (Figure 2, top left panel) and lower working memory span (Figure 2, top right panel). A similar pattern was observed for temporal-interval discrimination, as shown in Figure 3, although it was not significant. These findings indicate that participants who had no musical experience were more likely to have poor frequency discrimination, which were associated with generally poor verbal memory spans. Further research is required to determine whether this is also the case for temporal-interval discrimination. We should note that this group of poor performers had a similar contribution from each of the 3 schools that took part in this study, suggesting that this linkage is not an outcome of inter-school cultural/educational differences.

Table 2 shows that the pattern of correlations between auditory processing on the one hand and reading and reading-related measures on the other is indeed different in these two groups. While in the musically naïve group, the correlations with both measures of auditory processing are significant, they are not significant among individuals with more than one year of musical training. To assess the significance of this reduction in correlations, we calculated 95% confidence intervals (CI) for the significant correlations in the no-experience group using a bootstrapping procedure (CI columns in Table 2). Group differences were considered significant (and correlations were marked in bold) if the correlation for the more experienced group fell outside of this confidence interval. This analysis shows that the correlations between temporal-interval discrimination and the rest of the measures (cognitive skills, reading accuracy, memory span and working memory) were indeed significantly stronger in the group with no musical experience (the two leftmost columns in Table 2). Among musically naïve participants, partial correlations (controlling for the Raven scores) between temporal-interval discrimination thresholds and reading-related skills remained significant and similar in magnitude to the Pearson correlations (reading accuracy: -0.37, p = 0.022; memory span: r = -0.44, p = 0.005; working memory: r = -0.49, p = 0.002; phonological awareness: r = -0.36, p = 0.02). Therefore it appears that in this group, the correlations between auditory discriminations and reading-related skills are genuine and cannot be accounted for by general cognitive skills.

**Table 2:** Inter skill correlations in the sub-groups with (more than 12 months) and without musical training. Pearson correlations (r) and 95% confidence intervals (CI; for significant correlations).

	Frequency Discrimination			Temporal-interval Discrimination		
*Music experience (months*)	*0(n=40*)		*>12(n=26*)	*0(n=40*)		*>12(n=26*)
	r	CI	r	r	CI	r
Cognitive skill	-0.28		-0.06	-0.32*	-0.62-0.04	**0.06**
Reading accuracy	-0.31*	-0.56 0.08	-0.10	-0.38*	-0.62-0.02	**0.22**
Reading rate	0.01		0.19	0.12		0.03
Memory spans	-0.34*	-0.59-0.03	-0.34	-0.51**	-0.78-0.15	**-0.09**
Working memory	-0.37*	-0.62-0.07	-0.22	-0.56***	-0.73-0.32	**-0.09**
Phonological awareness	-0.32*	-0.56-0.02	-0.17	-0.42**	-0.68-0.11	-0.13

*p < 0.05; **p < 0.01; ***p < 0.001. Correlation values that are marked with bold typeface denote values that fall outside the CI of musically naïve participants.

## Discussion

We found that both frequency discrimination and interval discrimination are correlated with reading-related skills, as reported previously (e.g., [4,6,7,9,12,17]). These correlations remained significant when covariance with reasoning ability (assessed with Raven’s Matrices, a visual-spatial test) was controlled for. However, these correlations differed between children with different amounts of musical training. In "musically naïve” listeners there were significant correlations between frequency and temporal-interval discrimination on the one hand and reading accuracy, phonological awareness, verbal memory working memory on the other hand. These correlations reflect the abundance of individuals with poor auditory discrimination scores, who tend to have poor verbal memory scores. The proportion of individuals with poor auditory discrimination thresholds in the group with more than a year of musical training is much smaller. In this group, these correlations disappeared. While on average, this group had significantly lower frequency discrimination thresholds, and to a certain extent better temporal-interval discrimination, the distribution of thresholds mainly reflects a lack of very poor performers, rather than a clear over representation of very good performers. Together these findings suggest that a relatively small amount of musical training suffices to improve basic perceptual skills, particularly among initially poor performers, but does not immediately generalize to verbal and cognitive skills in the broader population of musically trained participants.

Although musically experienced children had better frequency discrimination thresholds even after their somewhat lower Raven scores were taken into account, the correlational design of the study makes it impossible to rule out an alternative interpretation for the relative scarcity of poor auditory processing in musically experienced children, namely that children with poorer auditory skills are simply less likely to take music lessons, although we find this less likely for the following reasons. First, musically experienced children in the current study were taking music lessons as part of an after-school activity in their schools. These after school programs do not screen participants for musical (or any other) aptitude. Therefore it seems less likely that children with poorer auditory skills were actively discouraged from taking music lessons, although they may have refrained from taking them for other reasons. Second, although consistent with the pre-existing differences account, children with any amount of musical experience had higher Raven scores than the musically naïve children, this was not the case for auditory processing. Had pre-existing differences in auditory processing, general ability or motivation been the cause of the observed differences between musically naïve and musically experienced children, we would expect auditory differences even between the naïve participants and those with 1-12 months of musical training. As shown in Figure 1, this was not the case.

### 1: Patterns of correlations

The present findings suggest that part of the controversy regarding the association between auditory processing and reading-related skills may be resolved by accounting for participants’ degrees of musical experience, which was not considered in most previous studies. This conclusion is consistent with the one previous study in which musical experience was considered as an independent variable in the analysis of the relationships between auditory processing (pitch, rhythm and timbre discrimination) and reading-related skills (word identification and phonological skills) [64]. Taken together, it thus seems that in “musically naïve” children of similar ages and reading instruction, auditory processing is consistently related to reading-related skills, and that the lack of associations reported in earlier studies (e.g., [20,23]) may result from testing samples with mixed musical experience, perhaps with a large representation of children with musical background. Indeed, in previous studies in which school-age children were tested on both frequency discrimination and reading-related tasks [1,17,26], the proportions of variance in reading-related scores accounted for by frequency discrimination ranged from 0 to 0.25 (i.e. correlations ranging from 0 to .5). The correlations observed in the present study (see Table 1), are consistent with those findings, with frequency discrimination statistically accounting for 4% of the variance in reading accuracy, 6% of the variance in phonological awareness and 11% of the variance in memory spans. Among the musically naïve children in the current study (Table 2), frequency discrimination accounted for 9%, 10%, 11% and 13% of the variance in word reading, phonological awareness, memory spans and working memory, respectively. Interestingly, those values are similar in magnitude to the contribution of pitch processing to reading as reported for pre-school children (9%) [9]. Assuming that younger children are less likely to receive formal music training, it might therefore be that those values reflect the ‘true’ unique contribution of frequency discrimination to reading.

Interestingly, the magnitude of the effect was even larger for interval discrimination, where thresholds in musically naïve participants accounted for more than 30% of the variability in working memory spans. Yet, a corresponding analysis for temporal-interval discrimination is currently not feasible because the relationships between temporal-interval discrimination and reading were not as intensively studied in school-age children (though see 1 for a similar finding in Spanish speaking children).

### 2: Dynamics of improvement in frequency versus temporal-interval discrimination

Adults with long and intensive musical training attain substantially better thresholds than those we measured [32-34]. Nevertheless the current data suggest that even relatively minimal musical experience is associated with better frequency discrimination. Although the current data are also consistent with the idea that even individuals with relatively brief (~ 1 year) experience with formal music lessons have better temporal-interval discrimination than musically naïve ones, this effect was more subtle and manifested in changes to the pattern of correlations between temporal-interval discrimination and reading-related skills, and not in a change to mean discrimination thresholds. Since previous studies, conducted among highly experienced musicians, suggest that they have better temporal-interval discrimination thresholds [34,35], genuine threshold improvements seems to require more than a few months of musical training. Several reasons could account for the different time courses of frequency and temporal-interval discrimination. First, temporal-interval discrimination might be more resilient to modification than frequency discrimination. This however seems unlikely given the outcomes of studies in which naïve listeners were trained on either frequency or temporal-interval discrimination [15,33], because in those studies the learning profiles on the two tasks did not drastically differ in terms of the number of training days or the total number of trials required for learning. Alternatively, the initial stages of formal music learning may place a greater emphasis on skills involving pitch than on those involving rhythm thereby resulting in faster changes in frequency than in temporal-interval discrimination.

### 3: Dynamics of generalization to reading-related language skills

Previous studies reported that musicians’ verbal memory spans are larger than those of non-musicians [50,51,60], and that years of practice are correlated with verbal memory. Other studies that assessed the relations between verbal memory and musical experience, also studied populations with longer periods of musical training [71,72]. The current data suggest that one year of formal music training of the type characteristic of most (Israeli) children, that is taking music lessons as an after school activity, is not enough to induce a significant generalization to non-auditory skills. Although musically experienced children tended to have higher memory scores than their musically naïve peers, the effect was small and we could not tease it apart from the effects of the somewhat higher general cognitive skills in the musically experienced group. The most likely interpretation of this finding is that longer and/or more intensive musical training is required to drive changes in verbal memory than in pitch discrimination. This interpretation is in line with findings of a longitudinal study where musically experienced children with an average experience of 2.5 years had better verbal memory than children with no musical experience. When those children were followed longitudinally, their memory scores continued to improve with further musical practice, but not if they stopped taking music lessons [50].

In contrast to the weak relationships between musical experience and memory among individuals with average or above auditory skills, we found that both memory spans and working memory were particularly poor among children with poor auditory discriminations (Figures 2, 3). These children were quite prevalent in the “musically naïve” group, but quite rare in the musically trained group. Assuming that the distribution of auditory skills was initially similar in the two groups, this finding suggests that children with initially poor auditory processing might gain the most from relatively small amounts of training, with perhaps a broader initial transfer to language related skills. An earlier study we conducted in individuals with dyslexia and poor working memory suggests that this may indeed be the case. In that study [15], individuals with initially poor auditory processing and poor working memory were trained on frequency and duration discrimination until their performance on these tasks reached that of their adequately reading peers. Their perceptual learning generalized to improved verbal working memory (adequate Digit backward), and both perceptual and memory gains remained stable for at least few months after the end of training, when they were re-tested. Importantly, a relatively short period of practice was sufficient, suggesting that perhaps, short practice is sufficient to drive working memory changes when initial performance is very poor. Since children with poor auditory processing may be less likely to receive formal music instruction (at least in places where music lessons are not mandatory), future studies should specifically target this population.

Taken together, these findings suggest that generalization may be more immediate among individuals with initially very poor performance, compared with individuals with better initial thresholds, where evidence for broader improvement is mainly found after years of practice. Perhaps this putative difference reflects different learning mechanisms. Improvement among very poorly performing individuals may stem from strengthening broader top-down mechanisms [73], maybe improving a more general ability to sustain auditory attention, which is probably not the bottleneck limiting performance of individuals with better thresholds.

## Conclusions

Although the correlational design of the current study makes it impossible to reach definitive conclusions regarding the causal contribution of musical training to cognition, it highlights two important issues. First, the pattern of correlations between perceptual and non-perceptual skills differs between musically naive individuals and those with even relatively short musical training, a factor that was not taken into account in earlier studies. Second, the changes we associate with musical experience are not simultaneous and uniform changes across domains, or across populations. Rather, we tentatively propose that musical training readily and rapidly generalizes to frequency discrimination, then to interval discrimination, and only subsequently to enhanced language and memory skills. Moreover, the degree of generalization may depend on initial auditory performance, being perhaps broader among initially poor performers. Further studies in which children are randomly assigned to receive (or not receive) musical training and then followed over time are required to test this proposal. Nevertheless, the present findings suggest that baseline auditory processing should be considered when attempting to use and evaluate music as a form of intervention.

## References

[1] Goswami U, Wang HL, Cruz A, Fosker T, Mead N et al. (2011) Language-universal sensory deficits in developmental dyslexia: English, Spanish, and Chinese. J Cogn Neurosci 23: 325-337. doi:10.1162/jocn.2010.21453. PubMed: 20146613. 20146613 10.1162/jocn.2010.21453

[2] Tallal P (1980) Auditory temporal perception, phonics, and reading disabilities in children. Brain Lang 9: 182-198. doi:10.1016/0093-934X(80)90139-X. PubMed: 7363063. 7363063 10.1016/0093-934x(80)90139-x

[3] Ziegler JC, Goswami U (2005) Reading acquisition, developmental dyslexia, and skilled reading across languages: a psycholinguistic grain size theory. Psychol Bull 131: 3-29. doi:10.1037/0033-2909.131.1.3. PubMed: 15631549. 15631549 10.1037/0033-2909.131.1.3

[4] De Weirdt W (1988) Speech perception and frequency discrimination in good and poor readers. Appl Psycholinguistics 9: 163-183. doi:10.1017/S0142716400006792.

[5] Witton C, Talcott JB, Hansen PC, Richardson AJ, Griffiths TD et al. (1998) Sensitivity to dynamic auditory and visual stimuli predicts nonword reading ability in both dyslexic and normal readers. Curr Biol 8: 791-797. doi:10.1016/S0960-9822(98)70320-3. PubMed: 9663387. 9663387 10.1016/s0960-9822(98)70320-3

[6] Reed MA (1989) Speech perception and the discrimination of brief auditory cues in reading disabled children. J Exp Child Psychol 48: 270-292. doi:10.1016/0022-0965(89)90006-4. PubMed: 2794857. 2794857 10.1016/0022-0965(89)90006-4

[7] Ahissar M, Protopapas A, Reid M, Merzenich MM (2000) Auditory processing parallels reading abilities in adults. Proc Natl Acad Sci U S A 97: 6832-6837. doi:10.1073/pnas.97.12.6832. PubMed: 10841578. 10841578 10.1073/pnas.97.12.6832 PMC18757

[8] Talcott JB, Witton C, McLean MF, Hansen PC, Rees A et al. (2000) Dynamic sensory sensitivity and children’s word decoding skills. Proc Natl Acad Sci U S A 97: 2952-2957. doi:10.1073/pnas.040546597. PubMed: 10688885. 10688885 10.1073/pnas.040546597 PMC16036

[9] Anvari SH, Trainor LJ, Woodside J, Levy BA (2002) Relations among musical skills, phonological processing, and early reading ability in preschool children. J Exp Child Psychol 83: 111-130. doi:10.1016/S0022-0965(02)00124-8. PubMed: 12408958. 12408958 10.1016/s0022-0965(02)00124-8

[10] Talcott JB, Witton C, Hebb GS, Stoodley CJ, Westwood EA et al. (2002) On the relationship between dynamic visual and auditory processing and literacy skills; results from a large primary-school study. Dyslexia 8: 204-225. doi:10.1002/dys.224. PubMed: 12455851. 12455851 10.1002/dys.224

[11] Foxton JM, Talcott JB, Witton C, Brace H, McIntyre F et al. (2003) Reading skills are related to global, but not local, acoustic pattern perception. Nat Neurosci 6: 343-344. doi:10.1038/nn1035. PubMed: 12652304. 12652304 10.1038/nn1035

[12] Banai K, Ahissar M (2004) Poor frequency discrimination probes dyslexics with particularly impaired working memory. Audiol Neuro Otol 9: 328-340. doi:10.1159/000081282. PubMed: 15467286. 10.1159/000081282 15467286

[13] Walker KM, Hall SE, Klein RM, Phillips DP (2006) Development of perceptual correlates of reading performance. Brain Res 1124: 126-141. doi:10.1016/j.brainres.2006.09.080. PubMed: 17069776. 17069776 10.1016/j.brainres.2006.09.080

[14] Boets B, Wouters J, van Wieringen A, De Smedt B, Ghesquière P (2008) Modelling relations between sensory processing, speech perception, orthographic and phonological ability, and literacy achievement. Brain Lang 106: 29-40. doi:10.1016/j.bandl.2007.12.004. PubMed: 18207564. 18207564 10.1016/j.bandl.2007.12.004

[15] Banai K, Hornickel J, Skoe E, Nicol T, Zecker S et al. (2009) Reading and subcortical auditory function. Cereb Cortex 19: 2699-2707. doi:10.1093/cercor/bhp024. PubMed: 19293398. 19293398 10.1093/cercor/bhp024 PMC2758683

[16] Huss M, Verney JP, Fosker T, Mead N, Goswami U (2011) Music, rhythm, rise time perception and developmental dyslexia: perception of musical meter predicts reading and phonology. Cortex 47: 674-689. doi:10.1016/j.cortex.2010.07.010. PubMed: 20843509. 20843509 10.1016/j.cortex.2010.07.010

[17] Grube M, Kumar S, Cooper FE, Turton S, Griffiths TD (2012) Auditory sequence analysis and phonological skill. Proc Biol Sci 279: 4496-4504. doi:10.1098/rspb.2012.1817. PubMed: 22951739. 22951739 10.1098/rspb.2012.1817 PMC3479813

[18] Bishop DV, Bishop SJ, Bright P, James C, Delaney T et al. (1999) Different origin of auditory and phonological processing problems in children with language impairment: evidence from a twin study. J Speech Lang Hear Res 42: 155-168. PubMed: 10025551. 10025551 10.1044/jslhr.4201.155

[19] Hulslander J, Talcott J, Witton C, DeFries J, Pennington B et al. (2004) Sensory processing, reading, IQ, and attention. J Exp Child Psychol 88: 274-295. doi:10.1016/j.jecp.2004.03.006. PubMed: 15203301. 15203301 10.1016/j.jecp.2004.03.006

[20] Mody M, Studdert-Kennedy M, Brady S (1997) Speech perception deficits in poor readers: auditory processing or phonological coding? J Exp Child Psychol 64: 199-231. doi:10.1006/jecp.1996.2343. PubMed: 9120381. 9120381 10.1006/jecp.1996.2343

[21] Rosen S (1999) A problem with auditory processing? Curr Biol 9: R698-R700. doi:10.1016/S0960-9822(99)80443-6. PubMed: 10508602. 10508602 10.1016/s0960-9822(99)80443-6

[22] Rosen S, Manganari E (2001) Is there a relationship between speech and nonspeech auditory processing in children with dyslexia? J Speech Lang Hear Res 44: 720-736. doi:10.1044/1092-4388(2001/057). PubMed: 11521767. 11521767 10.1044/1092-4388(2001/057)

[23] White S, Milne E, Rosen S, Hansen P, Swettenham J et al. (2006) The role of sensorimotor impairments in dyslexia: a multiple case study of dyslexic children. Dev Sci 9: 237-255; discussion 265-239 doi:10.1111/j.1467-7687.2006.00483.x. PubMed: 16669791. 16669791 10.1111/j.1467-7687.2006.00483.x

[24] McArthur GM, Ellis D, Atkinson CM, Coltheart M (2008) Auditory processing deficits in children with reading and language impairments: can they (and should they) be treated? Cognition 107: 946-977. doi:10.1016/j.cognition.2007.12.005. PubMed: 18262177. 18262177 10.1016/j.cognition.2007.12.005

[25] Papadopoulos TC, Georgiou GK, Parrila RK (2012) Low-level deficits in beat perception: neither necessary nor sufficient for explaining developmental dyslexia in a consistent orthography. Res Dev Disabil 33: 1841-1856. doi:10.1016/j.ridd.2012.04.009. PubMed: 22695074. 22695074 10.1016/j.ridd.2012.04.009

[26] Georgiou GK, Protopapas A, Papadopoulos TC, Skaloumbakas C, Parrila R (2010) Auditory temporal processing and dyslexia in an orthographically consistent language. Cortex 46: 1330-1344. doi:10.1016/j.cortex.2010.06.006. PubMed: 20678760. 20678760 10.1016/j.cortex.2010.06.006

[27] Banai K, Ahissar M (2006) Auditory processing deficits in dyslexia: task or stimulus related? Cereb Cortex 16: 1718-1728. PubMed: 16407480. 16407480 10.1093/cercor/bhj107

[28] Ahissar M, Lubin Y, Putter-Katz H, Banai K (2006) Dyslexia and the failure to form a perceptual anchor. Nat Neurosci 9: 1558-1564. doi:10.1038/nn1800. PubMed: 17115044. 17115044 10.1038/nn1800

[29] Ahissar M (2007) Dyslexia and the anchoring-deficit hypothesis. Trends Cogn Sci 11: 458-465. doi:10.1016/j.tics.2007.08.015. PubMed: 17983834. 17983834 10.1016/j.tics.2007.08.015

[30] Heath SM, Hogben JH, Clark CD (1999) Auditory temporal processing in disabled readers with and without oral language delay. J Child Psychol Psychiatry 40: 637-647. doi:10.1111/1469-7610.00480. PubMed: 10357169. 10357169

[31] Kraus N, Chandrasekaran B (2010) Music training for the development of auditory skills. Nat Rev Neurosci 11: 599-605. doi:10.1038/nrm2968. PubMed: 20648064. 20648064 10.1038/nrn2882

[32] Kishon-Rabin L, Amir O, Vexler Y, Zaltz Y (2001) Pitch discrimination: are professional musicians better than non-musicians? J Basic Clin Physiol Pharmacol 12: 125-143. PubMed: 11605682. 11605682 10.1515/jbcpp.2001.12.2.125

[33] Micheyl C, Delhommeau K, Perrot X, Oxenham AJ (2006) Influence of musical and psychoacoustical training on pitch discrimination. Hear Res 219: 36-47. doi:10.1016/j.heares.2006.05.004. PubMed: 16839723. 16839723 10.1016/j.heares.2006.05.004

[34] Banai K, Fisher S, Ganot R (2012) The effects of context and musical training on auditory temporal-interval discrimination. Hear Res 284: 59-66. doi:10.1016/j.heares.2011.12.002. PubMed: 22200608. 22200608 10.1016/j.heares.2011.12.002

[35] Rammsayer T, Altenmuller E (2006) Temporal information processing in musicians and nonmusicians. Music Percept 24: 37-47. doi:10.1525/mp.2006.24.1.37.

[36] Pantev C, Oostenveld R, Engelien A, Ross B, Roberts LE et al. (1998) Increased auditory cortical representation in musicians. Nature 392: 811-814. doi:10.1038/33918. PubMed: 9572139. 9572139 10.1038/33918

[37] Peretz I, Zatorre RJ (2005) Brain organization for music processing. Annu Rev Psychol 56: 89-114. doi:10.1146/annurev.psych.56.091103.070225. PubMed: 15709930. 15709930 10.1146/annurev.psych.56.091103.070225

[38] Musacchia G, Sams M, Skoe E, Kraus N (2007) Musicians have enhanced subcortical auditory and audiovisual processing of speech and music. Proc Natl Acad Sci U S A 104: 15894-15898. doi:10.1073/pnas.0701498104. PubMed: 17898180. 17898180 10.1073/pnas.0701498104 PMC2000431

[39] Bidelman GM, Krishnan A, Gandour JT (2011) Enhanced brainstem encoding predicts musicians’ perceptual advantages with pitch. Eur J Neurosci 33: 530-538. doi:10.1111/j.1460-9568.2010.07527.x. PubMed: 21198980. 21198980 10.1111/j.1460-9568.2010.07527.x PMC3059719

[40] Parbery-Clark A, Strait DL, Kraus N (2011) Context-dependent encoding in the auditory brainstem subserves enhanced speech-in-noise perception in musicians. Neuropsychologia 49: 3338-3345. doi:10.1016/j.neuropsychologia.2011.08.007. PubMed: 21864552. 21864552 10.1016/j.neuropsychologia.2011.08.007 PMC3445334

[41] Moreno S, Marques C, Santos A, Santos M, Castro SL et al. (2009) Musical training influences linguistic abilities in 8-year-old children: more evidence for brain plasticity. Cereb Cortex 19: 712-723. doi:10.1093/cercor/bhn120. PubMed: 18832336. 18832336 10.1093/cercor/bhn120

[42] Fujioka T, Ross B, Kakigi R, Pantev C, Trainor LJ (2006) One year of musical training affects development of auditory cortical-evoked fields in young children. Brain 129: 2593-2608. doi:10.1093/brain/awl247. PubMed: 16959812. 16959812 10.1093/brain/awl247

[43] Shahin AJ, Roberts LE, Chau W, Trainor LJ, Miller LM (2008) Music training leads to the development of timbre-specific gamma band activity. NeuroImage 41: 113-122. doi:10.1016/j.neuroimage.2008.04.082. PubMed: 18375147. 18375147 10.1016/j.neuroimage.2008.01.067 PMC4349687

[44] Forgeard M, Winner E, Norton A, Schlaug G (2008) Practicing a musical instrument in childhood is associated with enhanced verbal ability and nonverbal reasoning. PLOS ONE 3: e3566. doi:10.1371/journal.pone.0003566. PubMed: 18958177. 18958177 10.1371/journal.pone.0003566 PMC2570220

[45] Magne C, Schön D, Besson M (2006) Musician children detect pitch violations in both music and language better than nonmusician children: behavioral and electrophysiological approaches. J Cogn Neurosci 18: 199-211. doi:10.1162/jocn.2006.18.2.199. PubMed: 16494681. 16494681 10.1162/089892906775783660

[46] Morrongiello BA, Roes CL (1990) Developmental changes in children’s perception of musical sequences: Effects of musical training. Dev Psychol 26: 814-820. doi:10.1037/0012-1649.26.5.814.

[47] Virtala P, Huotilainen M, Putkinen V, Makkonen T, Tervaniemi M (2012) Musical training facilitates the neural discrimination of major versus minor chords in 13-year-old children. Psychophysiology 49: 1125-1132. PubMed: 22681183. 22681183 10.1111/j.1469-8986.2012.01386.x

[48] Butzlaff R (2000) Can music be used to teach reading? J Aesthet Educ 34: 167-178. doi:10.2307/3333642.

[49] Crncec R, Wilson SJ, Prior M (2006) No evidence for the Mozart effect in children. Music Percept 23: 305-317. doi:10.1525/mp.2006.23.4.305.

[50] Ho YC, Cheung MC, Chan AS (2003) Music training improves verbal but not visual memory: cross-sectional and longitudinal explorations in children. Neuropsychology 17: 439-450. doi:10.1037/0894-4105.17.3.439. PubMed: 12959510. 12959510 10.1037/0894-4105.17.3.439

[51] Roden I, Kreutz G, Bongard S (2012) Effects of a school-based instrumental music program on verbal and visual memory in primary school children: a longitudinal study. Front Psychol 3: 572 PubMed: 23267341. 23267341 10.3389/fpsyg.2012.00572 PMC3528082

[52] Bidelman GM, Gandour JT, Krishnan A (2011) Musicians and tone-language speakers share enhanced brainstem encoding but not perceptual benefits for musical pitch. Brain Cogn 77: 1-10. doi:10.1016/j.bandc.2011.07.006. PubMed: 21835531. 21835531 10.1016/j.bandc.2011.07.006 PMC3159732

[53] Wong PC, Skoe E, Russo NM, Dees T, Kraus N (2007) Musical experience shapes human brainstem encoding of linguistic pitch patterns. Nat Neurosci 10: 420-422. PubMed: 17351633. 17351633 10.1038/nn1872 PMC4508274

[54] Meyer M, Elmer S, Ringli M, Oechslin MS, Baumann S et al. (2011) Long-term exposure to music enhances the sensitivity of the auditory system in children. Eur J Neurosci 34: 755-765. doi:10.1111/j.1460-9568.2011.07795.x. PubMed: 21848923. 21848923 10.1111/j.1460-9568.2011.07795.x

[55] Elbert T, Pantev C, Wienbruch C, Rockstroh B, Taub E (1995) Increased cortical representation of the fingers of the left hand in string players. Science 270: 305-307. doi:10.1126/science.270.5234.305. PubMed: 7569982. 7569982 10.1126/science.270.5234.305

[56] Penhune V, Watanabe D, Savion-Lemieux T (2005) The effect of early musical training on adult motor performance: evidence for a sensitive period in motor learning. Ann N Y Acad Sci 1060: 265-268. doi:10.1196/annals.1360.049. PubMed: 16597774. 16597774 10.1196/annals.1360.049

[57] Hughes CM, Franz EA (2007) Experience-dependent effects in unimanual and bimanual reaction time tasks in musicians. J Mot Behav 39: 3-8. doi:10.3200/JMBR.39.1.3-8. PubMed: 17251166. 17251166 10.3200/JMBR.39.1.3-8

[58] Parbery-Clark A, Skoe E, Lam C, Kraus N (2009) Musician enhancement for speech-in-noise. Ear Hear 30: 653-661. doi:10.1097/AUD.0b013e3181b412e9. PubMed: 19734788. 19734788 10.1097/AUD.0b013e3181b412e9

[59] Bialystok E, Depape AM (2009) Musical expertise, bilingualism, and executive functioning. J Exp Psychol Hum Percept Perform 35: 565-574. doi:10.1037/a0012735. PubMed: 19331508. 19331508 10.1037/a0012735

[60] Chan AS, Ho YC, Cheung MC (1998) Music training improves verbal memory. Nature 396: 128. doi:10.1038/24075. PubMed: 9823892. 9823892 10.1038/24075

[61] Strait DL, Kraus N, Parbery-Clark A, Ashley R (2010) Musical experience shapes top-down auditory mechanisms: evidence from masking and auditory attention performance. Hear Res 261: 22-29. doi:10.1016/j.heares.2009.12.021. PubMed: 20018234. 20018234 10.1016/j.heares.2009.12.021

[62] Herholz SC, Zatorre RJ (2012) Musical training as a framework for brain plasticity: behavior, function, and structure. Neuron 76: 486-502. doi:10.1016/j.neuron.2012.10.011. PubMed: 23141061. 23141061 10.1016/j.neuron.2012.10.011

[63] Wan CY, Schlaug G (2010) Music making as a tool for promoting brain plasticity across the life span. Neuroscientist 16: 566-577. doi:10.1177/1073858410377805. PubMed: 20889966. 20889966 10.1177/1073858410377805 PMC2996135

[64] Tsang CD, Conrad NJ (2011) Music training and reading readiness. Music Percept 29: 157-163. doi:10.1525/mp.2011.29.2.157.

[65] Banai K, Yifat R (2011) Perceptual anchoring in preschool children: not adultlike, but there. PLOS ONE 6: e19769. doi:10.1371/journal.pone.0019769. PubMed: 21603614. 21603614 10.1371/journal.pone.0019769 PMC3095618

[66] Banai K, Yuval-Weiss N (2013) Prolonged development of auditory skills: A role for perceptual anchoring? Cogn Dev 28: 300-311. doi:10.1016/j.cogdev.2013.05.002.

[67] Deutsch A, Bentin S (1996) Attention factors mediating syntactic deficiency in reading-disabled children. J Exp Child Psychol 63: 386-415. doi:10.1006/jecp.1996.0055. PubMed: 8923752. 8923752 10.1006/jecp.1996.0055

[68] Wechsler D (1998) Wechsler Intelligence Scale for Children (R-95,1998 Israeli edition) - Manual. San Antonio: The Psychological Corporation.

[69] Raven J, Raven JC, Court JH (2000) Manual for Raven’s progressive matrices and vocabulary scales. Oxford: Oxford Psychologists Press.

[70] Weiss MW, Schellenberg GE (2011) Augmenting cognition with music. In: Segev I Markram H Augmenting cognition. Lausanne: EPFL Press pp. 103-125.

[71] Jakobson LS, Lewycky ST, Kilgour AR, Stoesz BM (2008) Memory for verbal and visual material in highly trained musicians. Music Percept 26: 41-55. doi:10.1525/mp.2008.26.1.41.

[72] Kraus N, Strait DL, Parbery-Clark A (2012) Cognitive factors shape brain networks for auditory skills: spotlight on auditory working memory. Ann N Y Acad Sci 1252: 100-107. doi:10.1111/j.1749-6632.2012.06463.x. PubMed: 22524346. 22524346 10.1111/j.1749-6632.2012.06463.x PMC3338202

[73] Ahissar M, Nahum M, Nelken I, Hochstein S (2009) Reverse hierarchies and sensory learning. Philos Trans R Soc Lond B Biol Sci 364: 285-299. doi:10.1098/rstb.2008.0253. PubMed: 18986968. 18986968 10.1098/rstb.2008.0253 PMC2674477

